# Starting with a degron: N-terminal formyl-methionine of nascent bacterial proteins contributes to their proteolytic control

**DOI:** 10.15698/mic2015.10.235

**Published:** 2015-10-05

**Authors:** R. Jürgen Dohmen

**Affiliations:** 1 Institute for Genetics, University of Cologne, Zülpicher Str. 47, D-50674 Cologne, Germany.

**Keywords:** N-end rule, formyl-methionine, acetylation, FtsH, proteasome, ubiquitin, quality control

Generally, the synthesis of proteins in bacteria as well as in eukaryotic mitochondria and chloroplasts starts with formyl-methionine (fMet). The formyl group of Met is subsequently removed by ribosome-associated peptide deformylase (PDF) during translational elongation. The function of the transient formylation of the initiator Met residue, beyond enhancing the interaction with translation initiation factors, has remained enigmatic. Varshavsky and colleagues now report, in the current issue of *Microbial Cell*, that fMet is a determinant of a novel N-terminal de-gradation signal (N-degron) that targets nascent proteins for degradation in *Escherichia coli* by a new branch of the bacterial N-end rule pathway, termed the fMet/N-end rule pathway 1.

The N-end rule refers to the fact, first discovered in 1986 2, that the identity of the N-terminal residue of an intracellular protein can strongly influence the rate of its *in vivo* degradation. The underlying proteolytic systems, termed the N-end rule pathways, are present in all organisms, from bacteria to mammals 2,3,4. The N-end rule pathway was originally discovered in *Saccharomyces cerevisiae* as a ubiquitin-dependent system that mediates the degradation of proteins bearing certain (“destabilizing”) N-terminal residues (N-degrons) by the 26S proteasome 5. This system is conserved from yeast to mammals, with differences in details 2. An N-end rule pathway was then also discovered in bacteria (*E. coli*), which lack the bona fide ubiquitin/proteasome system. In the ubiquitin-independent bacterial N-end rule pathway, the degradation of proteins bearing N-degrons is mediated by the ClpS adaptor protein that delivers N-end rule substrates to the proteasome-like ClpAP protease 6,7,8. Interestingly, ClpS has sequence and structural similarities to a substrate-binding site of the yeast N-recognin Ubr1, the ubiquitin ligase that targets and ubiquitylates N-end rule substrates, as well as to related ubiquitin ligases in mammals 4,9,10,11.

Detailed analyses of N-end rule pathways in various organisms, spearheaded by the group of Alexander Varshavsky, have led to an expansion of the N-end rule from its original version and to appreciation of great complexity of the underlying protein machines 2. Many N-terminal residues that are recognized by the N-end rule pathway as destabilizing ones would not be expected to become N-terminal in the absence of posttranslational cleavages of proteins by endoproteases such as caspases, calpains or separases. Specifically, Met-aminopeptidases (MetAPs), the ribosome-associated proteases that can cleave off the initially present N-terminal Met of a nascent protein, would do so only if the residue at position 2 is not larger than Val 2,12. As a result, most residues that are destabilizing in the N-end rule would not become N-terminal cotranslationally unless a specific method, such as the ubiquitin fusion technique, is employed to expose a given amino acid residue at the protein’s N-terminus 5. The originally discovered N-end rule pathway, now called the Arg/N-end rule pathway, is mediated by Ubr1 in yeast and by related ubiquitin ligases in mammals and plants. This pathway targets proteins that bear the N-terminal residues Arg, Lys, His, Leu, Phe, Tyr, Trp, Ile, Asn, Gln, Asp, Glu or Cys 2. As mentioned above, most of these residues become N-terminal through endoproteolytic cleavages of specific proteins by proteases other than MetAPs. The resulting substrates of the Arg/N-end rule pathway include C-terminal cleavage products of specific cohesin subunits in yeast 13, caspase-generated protein fragments in *Drosophila* and mammals 14,15, and neurodegeneration-associated protein fragments 16. In addition, it was recently found that the Arg/N-end rule pathway can also target substrates bearing unmodified N-terminal Met, if it is followed by a bulky hydrophobic residue 17.

Recent landmark studies by Varshavsky and colleagues revealed that this was still not the full N-end rule story. Specifically, it was found that N-terminal acetylation (Nt-acetylation) of proteins, often at N-terminal residues that are not recognized by the Arg/N-end rule pathway, creates a new kind of N-degrons (termed Ac/N-degrons), which are targeted by ubiquitin ligases (Doa10 and Not4 in yeast, and Teb4 in mammals) that are distinct from ligases of the Arg/N-end rule pathway 18,19,20. The resulting new branch of the N-end rule pathway was termed the Ac/N-end rule pathway. It was also shown that the activity of an Ac/N-degron is conditional in that it can be repressed, for example, through its steric shielding within a multisubunit protein complex 19 (Figure 1A). Given their conditional nature, Ac/N-degrons are thought to serve quality control and regulatory functions 19. The conditionality of Ac/N-degrons and their functions in quality control are likely to account for the fact that a large majority of eukaryotic proteins are cotranslationally and irreversibly Nt-acetylated by ribosome-associated Nt-acetylases 21.

**Figure 1:**
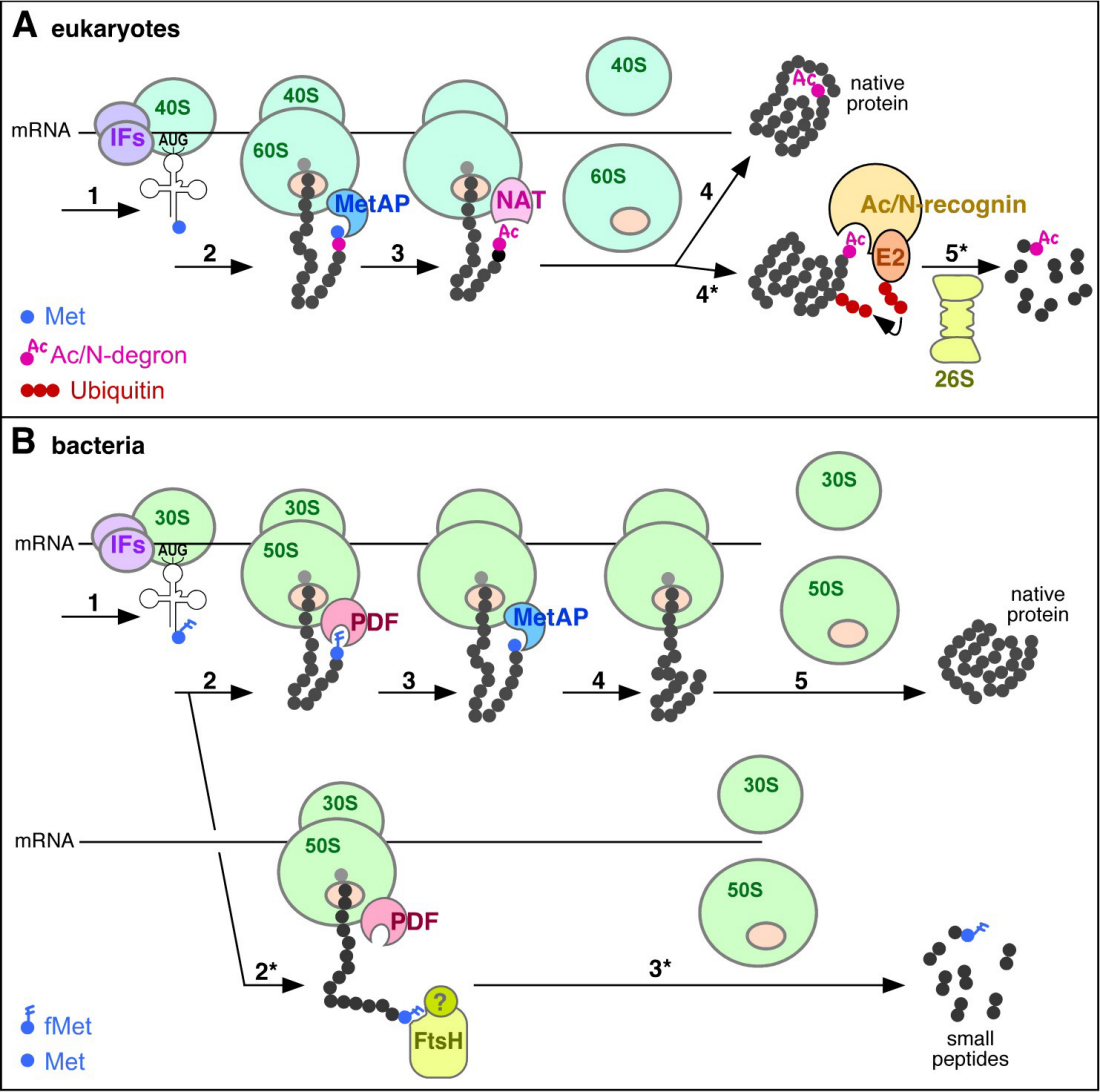
FIGURE 1: Role of N-terminal modifications in proteolytic targeting of nascent proteins. **(A) **Role of Nt-acetylation in the degradation of nascent proteins in eukaryotes. Step 1, translation initiation by a complex of the small ribosomal subunit (**40S**), initiation factors (**IFs**) and Met-tRNA; step 2, translation elongation after binding of the large ribosomal subunit (**60S**); step 3: removal of Met by Met-aminopeptidase (**MetAP**); step 4: N-terminal acetylation of the α-amino group by Nt-acetyltransferase (**NAT**), followed by the completion of translation, and the release of ribosomal subunits as well as of the completed, folded protein, with sterically shielded Nt-acetyl group. Alternatively, in step 5*, an Ac/N-degron that is not repressed (owing, for example, to an impaired folding of nascent protein or the absence of a cognate binding partner) is bound by an Ac/N-recognin (a specific ubiquitin ligase) in complex with an E2 ubiquitin-conjugating enzyme. The resulting targeted protein is polyubiquitylated and processively degraded by the 26S proteasome. **(B)** Possible role of N-terminal formyl-methionine (**fMet**) in the cotranslational quality control of nascent proteins in bacteria. Step 1, translation initiation by a complex of the small ribosomal subunit (**30S**), IFs and fMet-tRNA; step 2: translation elongation after binding of the large ribosomal subunit (**50S**); step 3, deformylation of fMet by the peptide deformylase (**PDF**) that transiently binds near the peptide exit tunnel of the ribosome; step 4: removal of deformylated Met by MetAP; step 5: completion of synthesis, followed by the release of ribosomal subunits and translated protein from mRNA. Alternatively, in step 2*, if the emerging nascent protein is not deformylated by PDF efficiently enough (for reasons mentioned in the main text) the fMet-bearing protein is recognized either directly by a protease (possibly by the FtsH protease) or by an adaptor (Ac/N-recognin) protein of unknown identity (indicated by a "?"), leading to processive degradation of the nascent protein in step 3*. Experimental data 1 suggest that the degradation via the fMet/N-end rule pathway occurs to a large extent cotranslationally as shown on the diagram.

The similarity of the formyl group of N-terminal fMet in bacteria and the acetyl groups as parts of Ac/N-degrons in eukaryotes led Varshavsky and colleagues to ask whether N-terminal fMet of bacterial proteins may also act as an N-degron 1. To address this question, the authors first performed pulse-chase assays in the presence of the deformylase (PDF) inhibitor actinonin and found that levels of larger polypeptides were disproportionately reduced, suggesting that they were lost owing to a higher rate of their degradation in the presence of actinonin. Starting from these more global effects caused by the inhibition of deformylation on protein levels, the identity of the relevant N-degron was investigated further using specific test proteins and appropriate controls. The results strongly suggested that inefficiently removed formyl groups of N-terminal fMet can selectively target proteins for degradation 1.

Based, in part, on these results, Varshavsky and colleagues propose a provocative and plausible hypothesis that fMet acts as an N-degron that serves as a cotranslational quality control sensor. This hypothesis is consistent with the fact that the action of PDF deformylase on a protein emerging from the ribosomal exit tunnel is confined to a narrow time window and is further constrained by conformational and sequence features of the nascent chain 1. PDF acts before the trigger factor (TF) chaperone binds to nascent chains of emerging proteins, and the binding by TF apparently takes place after the emerging polypeptide chain becomes longer than about 100 residues 22. If the emerging polypeptide is sufficiently flexible and if its N-terminal fMet is (stochastically) often exposed to solvent, the probability is high that PDF, which is bound to the ribosome close to exit tunnel 23, would deformylate the N-terminal fMet residue (Figure 1B). If, however, the conformational and/or sequence features of a nascent polypeptide slow down deformylation (this was experimentally mimicked by using test substrates with residues at position 2 that are suboptimal for fMet deformylation), the resulting time window may prove sufficient for the recognition of fMet as an N-degron and the ensuing degradation of a nascent protein 1 (Figure 1B). The observation that the levels of such proteins, in contrast to otherwise identical ones that are efficiently deformylated, were dramatically reduced after 1-min pulse labeling, suggested that fMet-mediated protein degradation is largely cotranslational 1.

What are the specific cellular components that mediate the fMet/N-end rule pathway? A very recent study showed that the proteolytic targeting of *E. coli* membrane-embedded YfgM protein depends on its N-terminal sequence and is mediated by the ATP-dependent protease FtsH 24. Varshavsky and colleagues suggest, based upon data in ref. 24, and their own preliminary data, that YfgM may be targeted by an fMet/N-degron. If so (this remains to be definitively verified), the data of the cited study 24 would identify FtsH as a protease that mediates the fMet/N-end rule pathway.

## CONCLUSION

In their hypothesis-driven study, Varshavsky and colleagues provide compelling (though still not definitive) evidence for the existence of an fMet-based bacterial N-degron and thus for a new function of the formyl modification of the fMet residue at the N-termini of virtually all nascent bacterial proteins 1. In addition, these findings provide a plausible explanation for earlier results, obtained through the use of the PDF inhibitor actinonin with plant chloroplasts. The new interpretation of these earlier data 25 suggests that fMet/N-degrons may be relevant to the cotranslational proteolytic control in eukaryotic organelles as well. Now that the first evidence for the fMet/N-end rule pathway has been produced, further studies are required to understand how fMet-bearing proteins are re-cognized and selected for degradation, possibly by the membrane-bound FtsH protease. The initial recognition of fMet may be mediated, for example, by an fMet binding site of the protease. Alternatively, the recognition of N-terminal fMet may involve a specific adaptor protein ana-logous to the ClpS N-recognin of the classical (ClpAP-mediated) bacterial N-end rule pathway. Yet another direction of future studies would address physiological conditions that influence the fMet-mediated protein degradation. The FtsH-mediated degradation of YfgM, for example, occurs in the stationary phase, whereas YfgM is relatively long-lived in the exponential growth phase 24. It would be also interesting to determine, for a broad range of bacterial proteins, exactly how the known sequence preferences of the PDF deformylase, the local folding properties of nascent polypeptide chains, and the co- or posttranslational steric shielding of N-terminal fMet influence the targeting of proteins by the fMet/N-end rule pathway.
